# Description of the New Species *Laccaria albifolia* (Hydnangiaceae, Basidiomycota) and a Reassessment of *Laccaria affinis* Based on Morphological and Phylogenetic Analyses

**DOI:** 10.3390/jof11010011

**Published:** 2024-12-27

**Authors:** Francesco Dovana, Roberto Para, Gabriel Moreno, Edoardo Scali, Matteo Garbelotto, Bernardo Ernesto Lechner, Luigi Forte

**Affiliations:** 1 Dipartimento di Bioscienze, Biotecnologie e Ambiente (DBBA), Campus Universitario “Ernesto Quagliariello”, Università degli Studi di Bari “Aldo Moro”, Via Orabona 4, 70125 Bari, Italy; luigi.forte@uniba.it; 2 Via Martiri di via Fani 22, 61024 Mombaroccio, Italy; r.para@alice.it; 3 Department of Life Sciences (Botany), Biology Building, University of Alcalá, 28805 Alcalá de Henares, Spain; gabriel.moreno@uah.es; 4 Department of Environmental Science, Policy and Management, University of California, 54 Mulford Hall, Berkeley, CA 94720, USA; edoardo_scali@berkeley.edu (E.S.); matteog@berkeley.edu (M.G.); 5 Laboratorio de Hongos Agaricales, Departamento de Biodiversidad y Biología Experimental, Facultad de Ciencias Exactas y Naturales, Universidad de Buenos Aires, Buenos Aires 1428, Argentina; bernardoelechner@gmail.com; 6 Instituto de Micología y Botánica (InMiBo), CONICET—Universidad de Buenos Aires, Buenos Aires 1428, Argentina

**Keywords:** *Agaricales*, phylogeny, taxonomy

## Abstract

*Laccaria* is a diverse and widespread genus of ectomycorrhizal fungi that form symbiotic associations with various trees and shrubs, playing a significant role in forest ecosystems. Approximately 85 *Laccaria* species are formally recognised, but recent studies indicate this number may be an underestimation, highlighting the need for further taxonomic studies to improve our understanding of species boundaries. This manuscript focuses on *Laccaria affinis*, originally described by Singer in 1967 as *Laccaria laccata* var. *affinis*, and details a comprehensive study of its morphological and molecular characteristics, including the examination of its holotype and recent collections from Italy and the United Kingdom. Our findings reveal significant micromorphological traits that enhance the original description. Phylogenetic analyses indicate that *L. affinis* occupies a distinct clade within Northern Hemisphere *Laccaria* species, although minimal genetic differences challenge its independence from *L. macrocystidiata*. Consequently, we propose that these two taxa be considered synonymous. This study not only contributes to the understanding of *Laccaria* diversity but also proposes the formal designation of an epitype for *L. affinis*, thereby providing a foundation for future research on this ecologically significant genus. Furthermore, a new species named *Laccaria albifolia* belonging to the “/Laccaria bicolor complex clade” is described here on the base of six collections from Italy and Spain.

## 1. Introduction

*Laccaria* Berkeley & Broome is a species-rich and widely distributed genus [1] of ectomycorrhizal fungi capable of establishing symbioses with numerous tree and shrub species [2]. According to He et al. [3], the genus *Laccaria* includes 85 described species, but the more recent description of numerous new *Laccaria* species from different parts of the world [4,5,6,7] highlights that this number is a gross underestimation of the actual number of *Laccaria* species. The uniformity of the morphological characters among many *Laccaria* taxa often requires molecular studies to clarify the relationships among taxa and to establish whether a taxon is new to science. The main aim of this manuscript is to carry out a study on *Laccaria laccata* var. *affinis* Singer, a species described by Singer [8] based on collections from the Bedgebury National Pinetum in Kent (England). Singer [8] placed this taxon within stirps *Laccata* as it is characterized by the presence of four-spored basidia, relatively small basidiospores up to 10.5 µm in length (ornamentation included) with up to 1.2 µm high spines, white basal mycelium, absence of violaceous tinges in the lamellae and white spore print. *Laccaria laccata* var. *affinis* is distinguished from *L. laccata* (Scop.) Cooke mainly by the more robust basidiomata (pileus > 3 cm in diameter), larger globose to subglobose spores (9.3–10.3 × (8.8)–9–10 µm vs. 8.5–9.5(10) × 6.7–8(9) in *var. laccata*) and larger cheilocystidia with variable shape [8]. Singer’s choice to consider it only a variety of *L. laccata* and not an independent species was in part influenced by the absence of sexual compatibility data, given the impossibility of culturing primary mycelia of monosporic origin. In 1983, Bon raised *L. laccata* var. *affinis* to the species level based on morphological differences with *L. laccata* and, in particular, based on the presence of globose spores in *L. affinis* (Singer) Bon [9]. Five years later, Migliozzi and Lavorato [10] described *Laccaria affinis* f. *macrocystidiata* Migl. & Lavorato as characterised by pink-red large basidiomata with large cheilocystidia. This latter taxon was elevated to the species rank by Pázmány [11], even if Mueller [2] had previously considered this taxon to be a synonym of *Laccaria laccata* var. *pallidifolia* (Peck) Peck. According to Contu’s monograph on the genus *Laccaria* in Italy, *L. affinis*, *L. laccata* and *L. macrocystidiata* are three independent species easily separable at the micromorphological level [12]. The third species would be separable from the previous ones due to the presence of large cheilocystidia and caulocystidia, as well as due to its growth range in the Mediterranean area [12]. Recently, Dovana et al. [13] conducted a morphological and molecular study on *L. macrocystidiata,* reporting a wide morphological variability within this taxon, particularly with regard to the shape and size of the spore ornamentations. In the same study, the independence of *L. macrocystidiata* from *Laccaria laccata* var. *pallidifolia* (based on reference collections from the USA reported in Osmundson et al. and Wilson et al. [14,15]) was shown based on phylogenetic studies of molecular data, but the relationship between *L. macrocystidiata* and *L. affinis* could not be resolved because the holotype of *L. affinis* had not yet been studied. The aims of the present study are as follows: (I) to study the holotype of *L. affinis*; (II) to describe recent collections of *L. affinis* and show coloured iconography of fresh collections of it and designate a representative sample from the type location; (III) to perform phylogenetic analyses in order to phylogenetically place *L. affinis* within *Laccaria*; and (IV) to describe, based on six collections obtained from thermophilic forests in Italy and Spain, a new *Laccaria* species, phylogenetically and morphologically close to *L. araneosa* H.J. Cho & Y.W. Lim.

## 2. Materials and Methods

### 2.1. Morphology

The macroscopic descriptions included in this study are based on fresh material collected in Italy, Spain and the UK. Descriptions of the micro-morphological characteristics are based on the study of fresh and dried material rehydrated in water, KOH or NH_4_OH, while other reagents used were Congo Red and Phloxin B. All measurements were conducted with an optical trinocular Nikon Eclipse E200. For ultramicroscopic studies, one lamella was placed on a 2 × 2 cm square of Whatman filter paper no. 2; the paper was folded into a packet to prevent the loss of spores and stapled shut, after which the packeted specimen was rehydrated in concentrated ammonium hydroxide (28–30%) for 30 min, dehydrated in aqueous ethanol (70%) for 30 min, fixed for two hours in pure ethylene glycol dimethyl ether (=1,2-dimethoxymethane), immersed in pure acetone for at least two hours, and finally critically point dried and sputtered with gold–palladium. This technique uses very little material (a portion of a lamella). Photographs of spores by SEM were obtained using a Zeiss-DSM 950 scanning electronic microscopy after critical point drying and sputtering [16]. A minimum of 30 basidiospores were measured for each collection. Basidiospore dimensions (measured without ornamentation) are expressed as (a) b–c–d (e), where (a) = minimum value, b = average—standard deviation, c = average, d = average + standard deviation, and (e) = maximum value. [x/y/z] indicates that x spores from y samples and z collections were measured altogether. Q indicates the quotient of length and width of the basidiospores in the side view. Basidia were measured without sterigmata.

### 2.2. Molecular Phylogeny

Genomic DNA was extracted from dried fragments of fourteen recently collected specimens using either the CTAB method as described by Doyle and Doyle [17], the NaOH DNA extraction method [18], or the Qiagen Mini Kit following the manufacturer’s instructions. The internal transcribed spacer (nrITS) was amplified with primers ITS1F/ITS4 [19,20], a portion of large subunit ribosomal ribonucleic acid (nrLSU) region with primers LR0R/LR5 [21], the RNA polymerase II subunit (*RPB2*) with primers used by Matheny [22], and the translation elongation factor 1-alpha (*TEF1-α*) following the protocols reported in Rehner & Buckley and Sheedy et al. [23,24]. The PCR products were purified and sequenced using Sanger’s method. The holotype of *L. affinis* was sequenced for the ITS region using an NGS approach. Due to repeated failures with other methods, the ITS region of the *L. affinis* holotype was sequenced using the ITS1-F and ITS-2 primer combination to target the ITS-1 region and the 5.8S-Fun and ITS4-Fun primer combination to target the ITS-2 region. These primers consisted of the published primer sequences plus one, two, three, four or five random nucleotides to act as spacers, as well as stubs, which complimented our Illumina adapters [25]. The concentration and quality of amplicons to be sequenced through Illumina-based High Throughput Sequencing were determined by gel imaging and Qubit measurements. Amplicon cleanup was performed using the Ampure cleanup kit. A second nested PCR was employed to attach Illumina adapters to the cleaned amplicon. Sequencing was then conducted using the NextSeq P1 300PE Illumina platform.

To extract the ITS sequences from the NGS data, we adopted two bioinformatics approaches, one based on the clustering of Amplicon Sequence Variants (ASVs) and another one based on the alignment of raw reads to the genome of *L. bicolor*. The genome file was retrieved from GenBank, where it is deposited under the accession number GCA_000143565.1. Sequence data were preprocessed with HTStream to trim the adapter, generate paired-end consensus sequences, trim low-quality ends, and remove low-quality paired-end reads. Denoising and ASVs were obtained using DADA2 (v3.16) [26]. Fungal ASVs were classified using the general fasta release of the UNITE database (v10.0) [27], which contains a total of 232,937 eukaryotic representative sequences. All the taxa that were not classified as *Laccaria* spp. were filtered out. The second method involved mapping post-processed paired ends read to the *L. bicolor* genome. Hisat2 (v2.2.0) [28] was used to create a genome index and map paired-end reads. Mapped reads were converted to a multi-FASTA file using Samtools utilities (v1.21) [29]. The resulting file contained all reads successfully mapped to the *L. bicolor* genome, among which nrITS sequences were identified using the UNITE database.

### 2.3. Sequence Alignment, Dataset Assembly and Phylogenetic Analysis

The sequences obtained in this study were checked and assembled using Geneious vs. R 11.1.5 [30] and compared to those available in the GenBank database (https://www.ncbi.nlm.nih.gov/genbank/) (accessed on 25 May 2024) and UNITE (accessed on 25 May 2024) databases (https://unite.ut.ee/) by using the BLASTN algorithm [31]. The nrITS, nrLSU, *RPB2* and *TEF1-α* datasets include sequences of *Laccaria* from the Northern Hemisphere and tropical regions. *Laccaria ambigua* K. Hosaka, A.W. Wilson & G.M. Mueller, reported as basal to the rest of the genus *Laccaria* in phylogeny proposed by Wilson et al. [15], was used as an outgroup. The nrITS, nrLSU, *RPB2* and *TEF1-α* sequences were independently aligned using MAFFT v 7.017 [32] with the E-INS-i algorithm. In order to generate a phylogenetic tree of the various taxa studied, Maximum Likelihood (ML) was inferred with IQ-TREE 2 [33]. The best models were selected using ModelFinder [34]. The SH-like approximate likelihood ratio test (with 1000 replicates) and ultrafast bootstrap approximation (UFB) (1000 replicates) [35] were used to evaluate the reliability of clades. The Bayesian inference (BI) was performed with MrBayes vs.3.2 [36] in the CIPRES Science Gateway [37]. Partition Finder 2 [38] was used to estimate the best partitioning schemes and evolution models for each subset with the MrBayes option. GTR+G+I was selected for each partition, and two independent analyses of four MCMC chains were run for 400 million generations, sampling trees every 1000 generations. The first 25% of trees were discarded as “burn-in”, and for the remaining trees, a majority rule consensus tree was computed to obtain estimates for Bayesian posterior probabilities (BPP). Significant support is considered to be ≥80% for the SH-aLRT test, ≥95% for ultrafast bootstrap support in the ML analysis, and ≥0.95 for BPP values in the Bayesian analysis. The list of sequences used in our dataset is given in Table S1 and includes *Laccaria* sequences selected based on previous molecular studies and the results in BLASTN [4,5,6,7,13,14,15,39,40,41,42,43,44,45,46,47,48,49,50,51,52,53,54,55,56,57,58,59,60,61,62,63,64,65].

## 3. Results

### 3.1. Molecular Phylogeny

**Figure 1:**
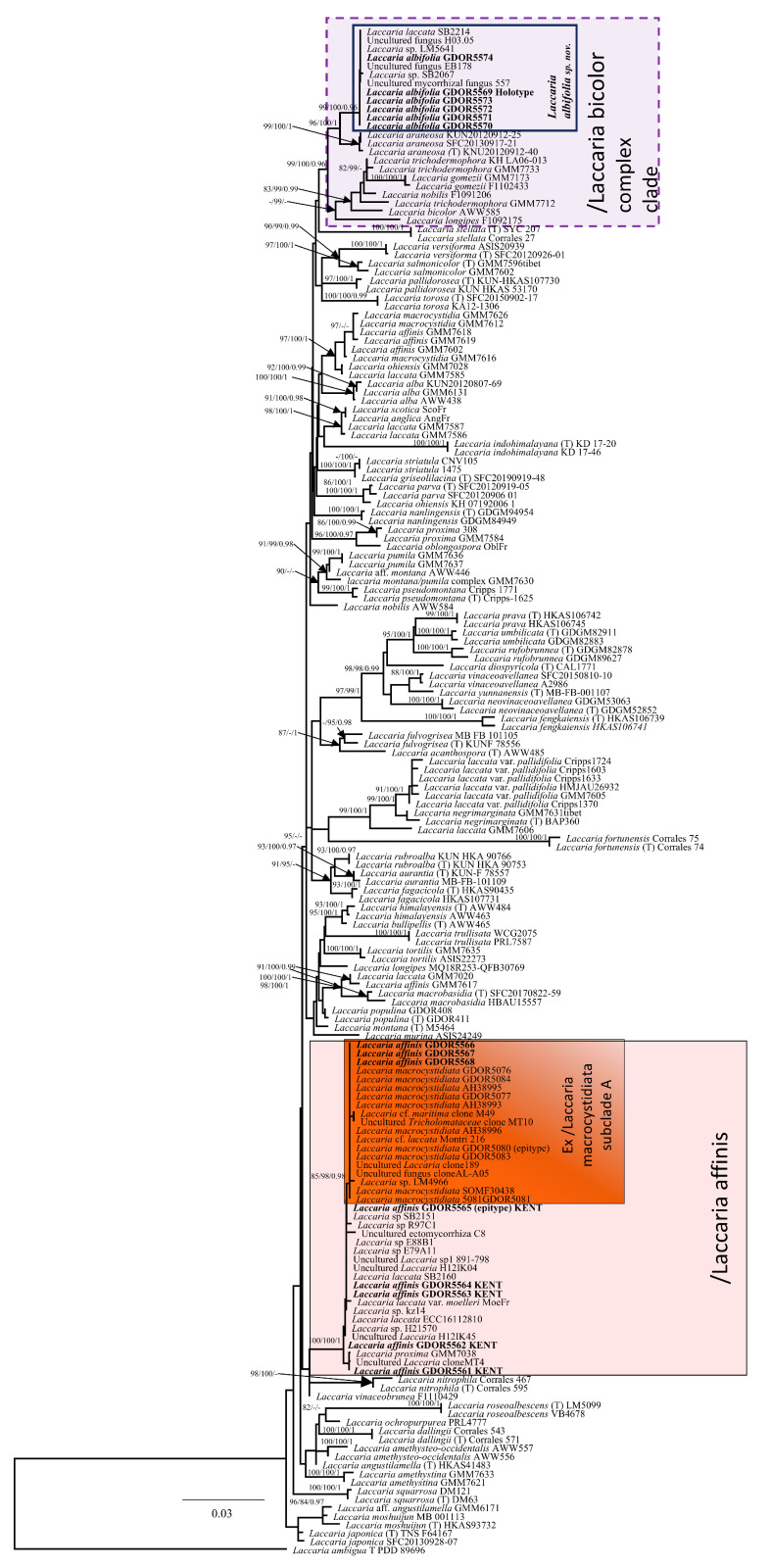
Maximum Likelihood phylogram obtained from the concatenated alignment of the nrITS, nrLSU, TEF1-α and RPB2 loci of selected *Laccaria* species from the northern hemisphere. *Laccaria ambigua* (T PDD 89696) was used as an outgroup taxon. Values above or below branches indicate bootstrap proportions SH-aLRT support ≥ 80%/ultrafast bootstrap support ≥ 95%/Bayesian posterior probabilities ≥ 0.95.

Fourteen nrITS, three nrLSU, two *RPB2* and two *TEF1-α* sequences of *Laccaria* were newly generated for this study (see Table S1). Despite several attempts, we were unable to obtain any *Laccaria* sequences from the *L. affinis* holotype. The nrITS, nrLSU, *RPB2* and *TEF1-α* combined datasets comprised 3285 characters and consisted of 178 lines. Both Bayesian and Maximum Likelihood analyses produced the same tree topology; therefore, only the ML tree with SH-aLRT, UFB and BPP values is shown (Figure 1). The four collections of *L. affinis* from locus classicus (Bedgebury National Pinetum in the UK) and a fifth collection from a location close to the type locality (associated with broadleaf trees) are placed within the clade previously designated as/L. macrocystidiata in Dovana et al. (2021) [13] (SH-aLRT = 100, UFB = 100, BPP = 1), which in this study is renamed/L. affinis. The samples previously named *L. macrocystidiata* in Dovana et al. [13] from Greece and Italy (including the epitype of *L. macrocystidiata*) and three new collections (originally identified as *L. macrocystidiata*) from Italy fall within/L. affinis clade in a supported subclade previously named “/L. macrocystidiata subclade A” in Dovana et al. [13]. The nrITS sequences belonging to/L. macrocystidiata subclade A and those of *L. affinis* from the type locality differ by one indel and one position.

In our phylogenetic analysis, six collections of *L. albifolia* from this study, clustered with six other sequences retrieved from GenBank in a well-supported clade (SH-aLRT = 99, UFB = 100, BPP = 0.96) named/L. albifolia. All 12 sequences above are placed within the /Laccaria bicolor clade (following Wilson et al. [15]) (SH-aLRT = 99, UFB = 100, BPP = 0.96), a clade that includes sequences of *L. araneosa*, *L. bicolor*, *L. gomezii*, *L. longipes*, *L. nobilis* and *L. trichodermophora*.

### 3.2. Taxonomy

*Laccaria affinis* (Singer) Bon, Docums Mycol. 13 (no. 51): 49 (1983) Figure 2, Figure 3, Figure 4, Figure 5, Figure 6 and Figure 7.

**Figure 2:**
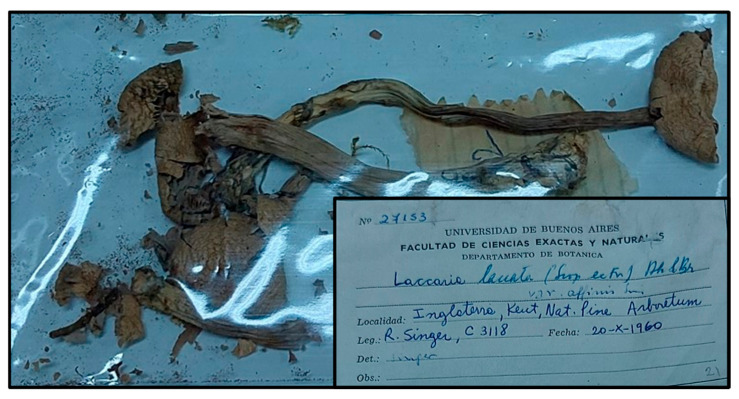
Specimen from the holotype of *L. affinis* (R. Singer, C3118) and label.

Basionym: *Laccaria laccata* var. *affinis* Singer, Bull. trimest. Soc. mycol. Fr. 83: 111 (1967);

=*Laccaria affinis* f. *macrocystidiata* Migl. & Lavorato, *Micol*. *Ital*. XVII(2): 6 (1988);

=*Laccaria affinis* var. *sardoa* M. Bon & Contu in *Doc. Mycol*. fasc. 59: 53 (1985) [based on morfological data];

=*Laccaria macrocystidiata* (Migl. & Lavorato) Pázmány, Zeitschrift für Mykologie 60 (1): 8 (1994);

=*Laccaria macrocystidiata* var. *longispinosa* Contu, *Bollettino del Gruppo Micologico “G. Bresadola”* 46 (1): 21 (2003);

Observations on “*Laccaria laccata* var. *affinis* holotype” (Figure 2).

The analysis was conducted on small fragments to preserve the integrity of the holotype specimen.

**Figure 3:**
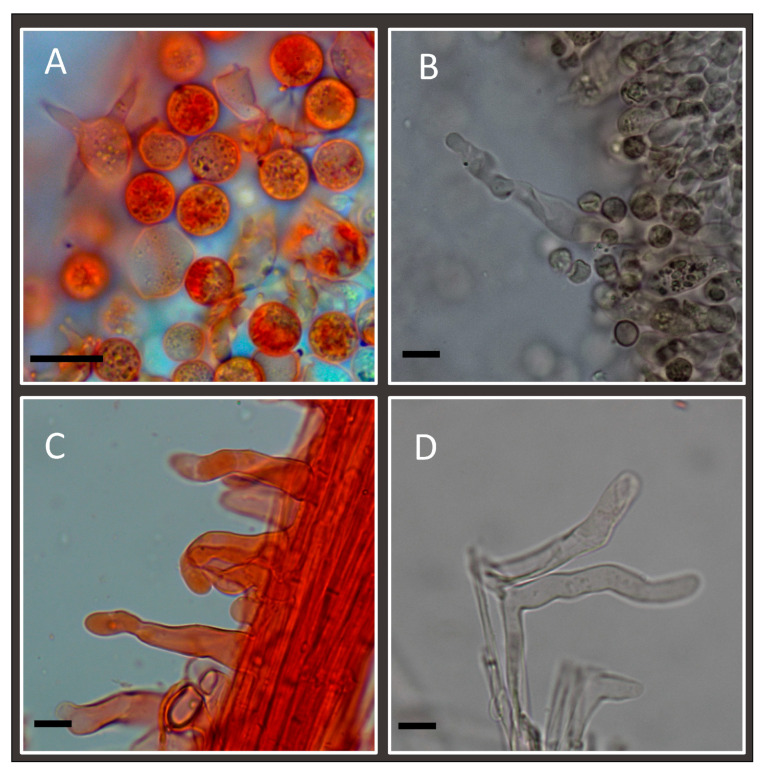
*Laccaria affinis* (Holotype): (**A**) basidia and basidiospores; (**B**) one of the rare cheilocystidia observed; (**C**,**D**) caulocystidia. Scale bar: 10 µm.

**Figure 4:**
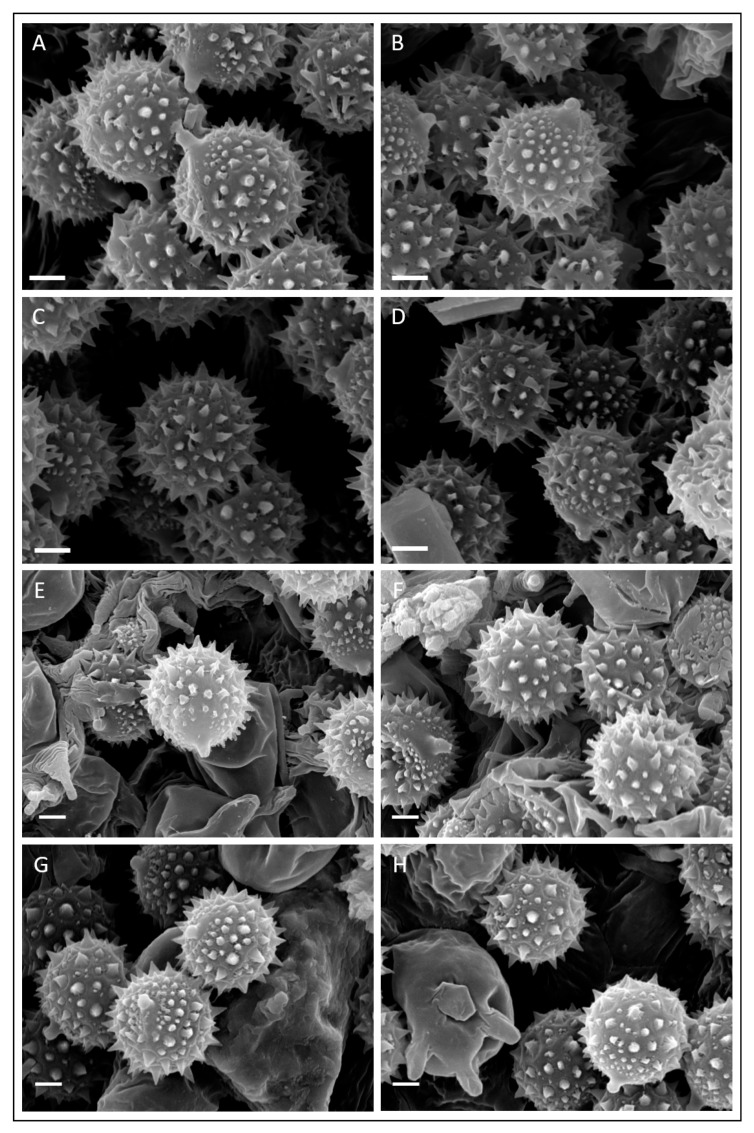
Basidiospores of *Laccaria affinis* (SEM photographs): (**A**–**D**) Holotype. (**E**,**F**) Collection GDOR_5562. (**G**,**H**) Collection GDOR_5561. Scale bars: 2 µm.

Spores (6.1–)7.2–7.8–8.5(–9.2) × (6.1–)7.1–7.7–8.4(–9.1) μm, Q = (0.93–)0.97–1.01–1.06(–1.12); many spores do not show spines under optical microscope (Figure 3A). Under SEM microscope, spores with conical-shaped spines, up to 1.5 µm high and with a base up to 1 µm wide (Figure 4). The spores of the holotype of *L. affinis* are identical to those from other collections of this taxon examined in this study collected in Kent. Cheilocystidia are very rare, variable in size and up to 66 × 12 µm (Figure 3B). Basidia is four-spored. Stipitipellis is a cutis with cystidia variable in shape and terminal cells up to 70 μm long (Figure 3C,D).

**Figure 5:**
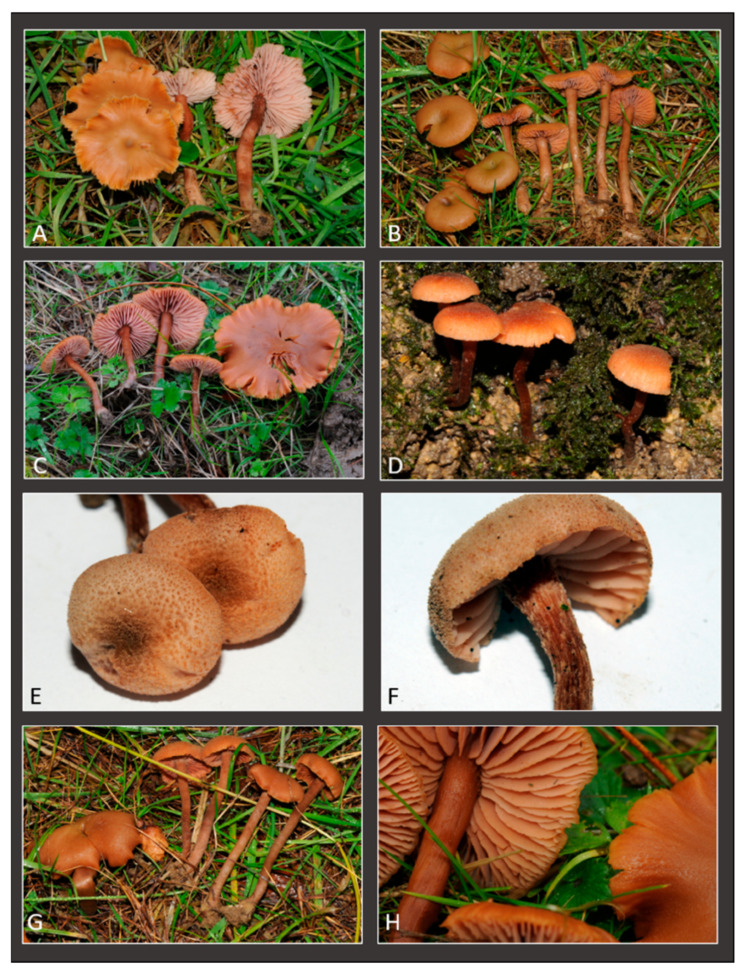
*Laccaria affinis* collected in Bedgebury National Pinetum (United Kingdom). Fresh basidiomata: (**A**) Collection GDOR_5562; (**B**) Collection GDOR_5563; (**C**) Collection GDOR_5564; (**D**–**F**) Collection GDOR_5561; (**G**,**H**) Collection GDOR_5565 (epitype).

**Figure 6:**
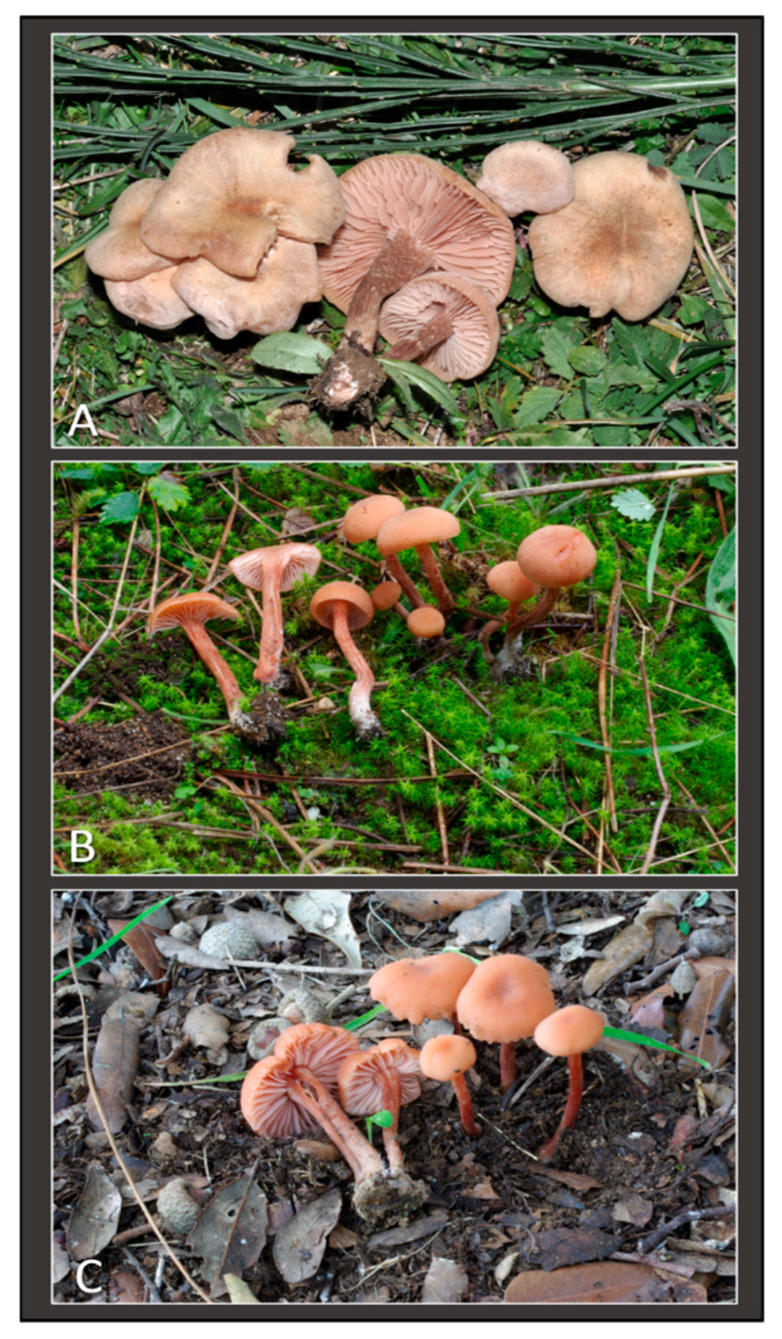
Italian collections of *Laccaria affinis* belonging to “subclade A”. Fresh basidiomata: (**A**). Collection GDOR_5567 (**B**). Collection GDOR_5566 (**C**). Collection GDOR_5568.

**Figure 7:**
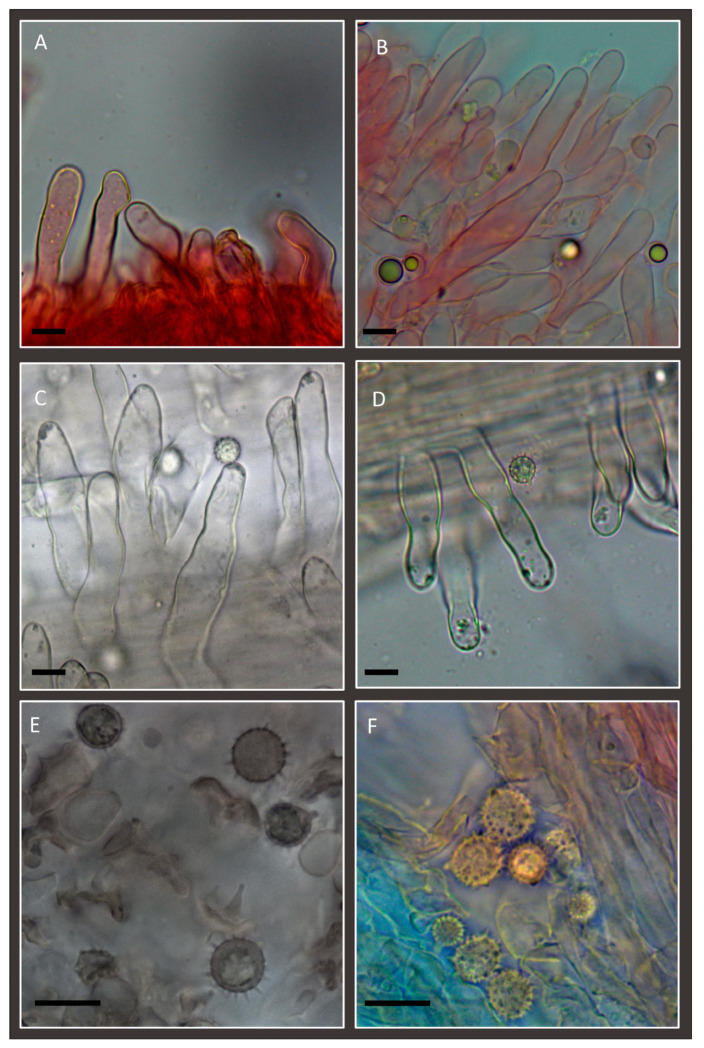
*Laccaria affinis*. (**A**,**B**) Cheilocystidia. (**C**,**D**) Caulocystidia. (**E**,**F**) Spores. Scale bars: 10 µm. (**A**,**C**–**F**) Collection GDOR_5565 (epitype). (**B**) Collection GDOR_5564.

Morfological description of *L. affinis* (Figure 4, Figure 5, Figure 6 and Figure 7):

Pileus 20–80 mm diam., at first hemispheric then convex to plano-convex, generally with a central depression, rarely striate, generally hygrophanous, generally covered with fine squamules or tufts (not present on pileus of some specimens collected under *Pinus* L. sp. and after heavy rain), pale brown, orange to orange-brown, pallescent to white or cream at centre upon drying. Lamellae emarginate, sometimes with a decurrent tooth, up to 13 mm broad, pale flesh-pink when young, later sordid pink-brown or concolourous with pileus. Stipe 30–150 × 3–12 mm, cylindrical, occasionally wider at the base, solid to fistulose when young, orange-brown or brown, generally darker than pileus, sometimes fibrillose-striate, white furfuraceous in the upper part, with white basal tomentum. Context hygrophanous, similar to or lighter than basidioma surface. Smell fungoid to herbaceous-fungoid. The taste is mild. The spore print is white.

Basidiospores (6.0–)7.7–8.6–9.4(–11.5) × (6.0–)7.6–8.5–9.3(–11.1) μm, Q = (0.93–)0.98–1.01–1.04(–1.25), avl × avw = 7.8–9.4 × 7.7–9.2 μm, avQ = 1.0–1.05, globose to subglobose, with spines variable in shape and dimension, from pyramidal to finely conical, up to 2.5 μm in length after rehydration. Basidia 25–45 × 10–16 μm, clavate, rarely flexuose, hyaline, 4-spored; sterigmata up to 9 μm long. Cheilocystidia 35–150 × 4–16 μm (cheilocystidia exhibit variability in size and shape across different basidiomata and even within the same basidioma, they are difficult to rehydrate in some dried specimens), cylindrical to flexuous, fusiform, rarely coralloid, generally obtuse, sometimes tapered towards the apex, colourless. Stipitipellis is a cutis formed by cylindrical hyphae, 3–6 μm wide, with frequent caulocystidia, either scattered or clustered. Caulocystidia 10–140 × 6–16 μm, variable in shape and size, ranging from clavate and cylindrical to flexuous. Pileipellis is a cutis made up of radially arranged cylindrical hyphae, often featuring uplifted bundles of hyphae; terminal elements resembling cheilocystidia, sometimes bifurcate. Clamp connections are present in all tissues.

Specimens examined:

United Kingdom: Kent, Bedgebury, broadleaf forest with a predominance of beech and oak, 08 November 2019, F. Dovana, GDOR_5561; Kent, Bedgebury, Bedgebury National Pinetum, under *Pinus* sp., 23 November 2019, leg. F. Dovana, GDOR_5562; ibidem, GDOR_5564; ibidem, under *Pinus* sp. and *Larix laricina*, GDOR_5563. Italy: Toscana, Livorno, loc. Rimigliano di San Vincenzo, forest with *Pinus pinaster* Aiton and *Quercus ilex* L., 14 December 2004, leg. R. Para, GDOR_5566; Calabria, Cosenza, loc. Serra di Buda di Acri, under *Pinus nigra* J.F.Arnold subsp. *laricio* Palib. ex Maire and *Castanea sativa* Mill., leg. C. Lavorato and M. Rotella, 24 November 2014, GDOR_5567; Toscana, Grosseto, Monterotondo Marittimo, loc. Torrente Milia, R. Para, GDOR_5568.

Epitype: MBT 10023645, designated here. GREAT BRITAIN: Kent, Bedgebury National Pinetum, under *Pinus* spp. and *Larix* sp., 23 November 2019, leg. F. Dovana, GDOR_5565.

**Figure 8:**
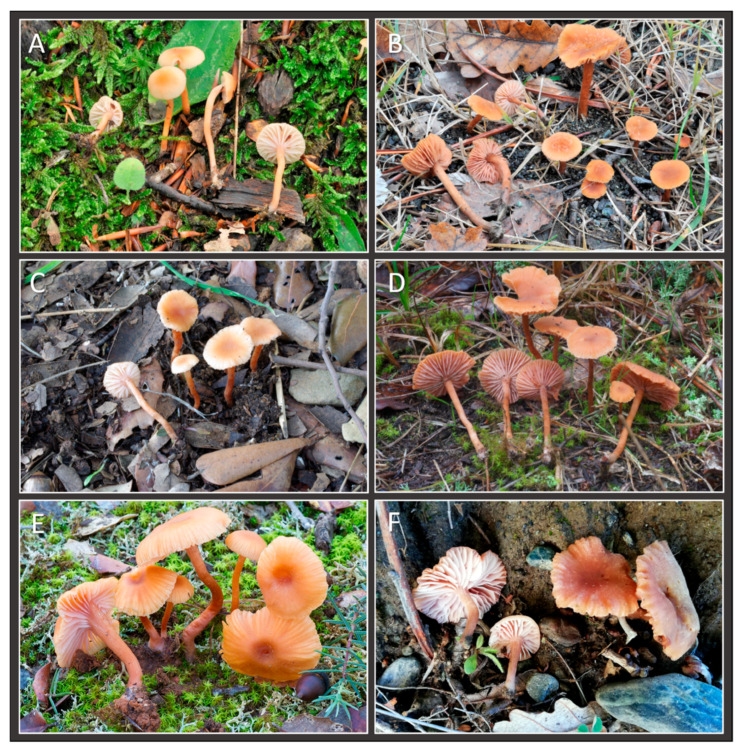
*Laccaria albifolia* basidiomata in situ: (**A**) GDOR_5569 (holotype); (**B**). GDOR_5570; (**C**) GDOR_5572; (**D**) GDOR_5573; (**E**). GDOR_5574; (**F**) GDOR_5571.

**Figure 9:**
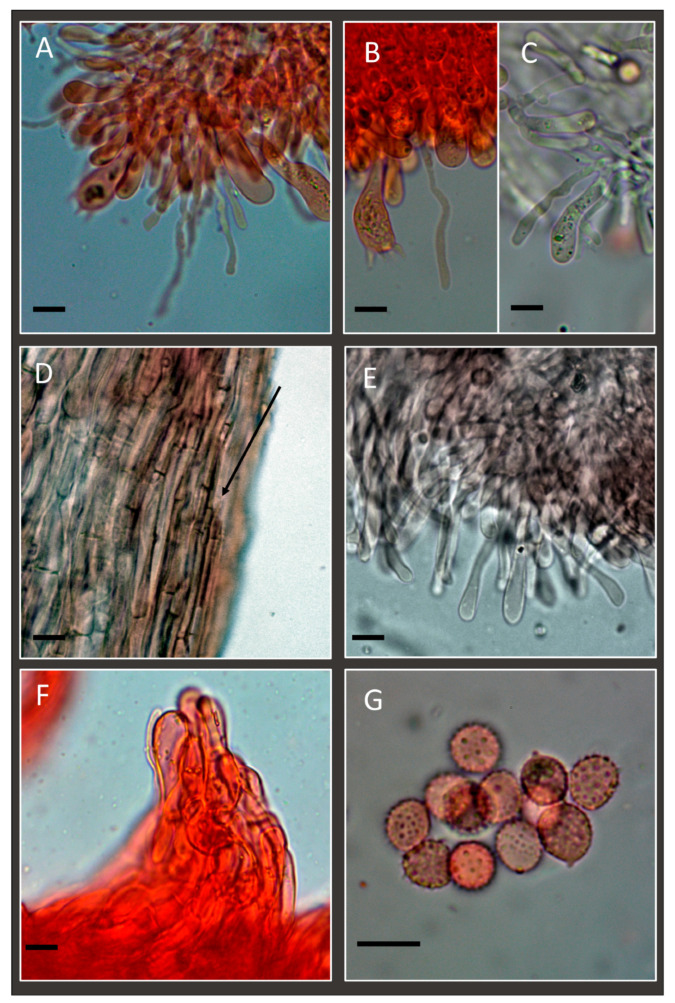
*Laccaria albifolia*: (**A**) Cheilocystidia (GDOR_5569 (holotype)). (**B**,**C**) Pleurocystidia. (**D**) stipitipellis; the arrow indicates the hyphae of the stipitipellis. (**E**) terminal elements of pileipellis at the edge of the pileus. (**F**) pileipellis with a perpendicular fascicle of hyphae. (**G**) Basidiospores. (**A**,**B**,**D**–**G**) in congo red; (**C**) in water. Scale bars: 10 µm.

**Figure 10:**
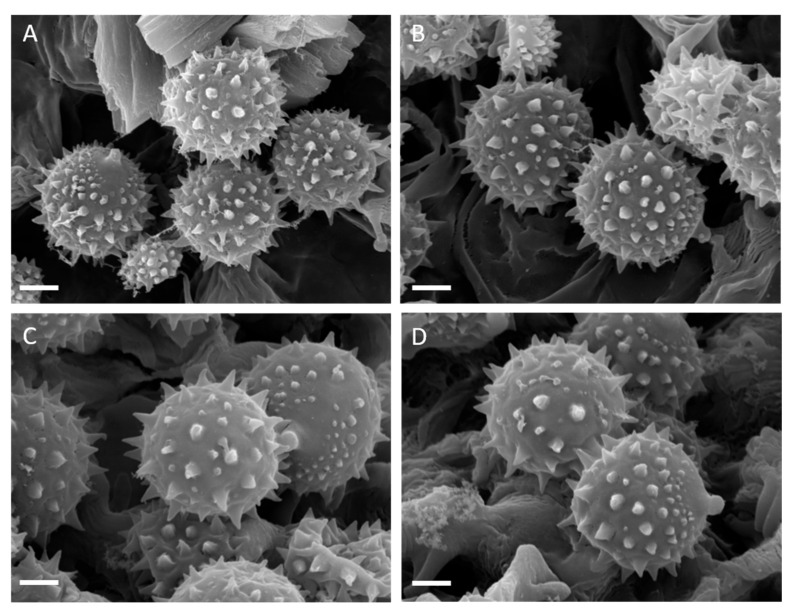
*Laccaria albifolia*. Spores (SEM photographs): (**A**,**B**) Collection GDOR_5569 (holotype). (**C**,**D**) Collection GDOR_5570. Scale bars: 2 µm.

*Laccaria albifolia* Dovana & Para, sp. nov. Figure 8, Figure 9 and Figure 10.

MB 856856. Etymology: albifolia—because of the whitish lamellae in typical specimens.

*Diagnosis*: Differing from *Laccaria araneosa* in its geographic distribution, white gills in the early stages of development, and the presence of cheilocystidia and pleurocystidia. Holotype: ITALY: Alessandria, Bosio, Capanne di Marcarolo, under *Fagus sylvatica* L., *Castanea sativa* Mill. and *Quercus* sp., 06 October 2010, leg. F. Dovana, GDOR_5569 (GenBank, nrITS: PQ642680, nrLSU: PQ642694, *TEF1-α*: PQ653979).

Pileus 5–30 mm in diameter, at first hemispherical, plano-convex or convex when young, then applanate to plano-concave, without umbo, glabrous, translucent-striate; orange (Munsell: 5YR7/8–12, 5YR6/6–10), hygrophanous, paler at margin (often whitish); margin deflexed to inflexed, undate to crenate. Lamellae emarginate to sinuate, rarely decurrent, with 1–3 lamellulae, whitish, sometimes subconcolorous with pileus with age. Stipe 15–60 × 2–4 mm, generally equal, sometimes slightly enlarged toward the base, straight to undulating, glabrous, minutely fibrillose, rubbery, orange, same colour as the centre of the pileus, or darker. Basal tomentum white. Context white or pale orange. Smell and taste indistinct.

Basidiospores [180, 6, 6] (6.2–)7.7–8.7–9.6(–10.4) × (6.2–)6.9–7.6–8.3(–9.4) μm, avl × avw = 7.7–9.3 × 7.1–8.0 μm, Q = (1.00–)1.06–1.15–1.24(–1.40), avQ = 1.09–1.19, globose to ellipsoid, with conical spines (up to 1.2 μm in length and less than 1 μm in wide at base). Basidia 38–65 × 10–15 µm, four-spored, clavate to flexuose. Cheilocystidia scattered to clumped, 17–80 × 2–4 μm, filamentous, subclavate to irregularly flexuose, thin-walled, hyaline. Pleurocystidia are mainly scattered, similar to cheilocystidia. Stipitipellis a cutis of 2–4 μm wide cylindrical hyphae; caulocystidia not observed. Pileipellis is a cutis of cylindrical hyphae, often interwoven with scattered perpendicular fascicles of hyphae; terminal elements of hyphae generally narrowly clavate. Lamellar trama regular to subregular. Clamp connections are present in all tissues.

Additional collections examined: Italy: Piemonte, Alessandria, Tagliolo Monferrato, loc. Colma (Parco Naturale delle Capanne di Marcarolo), forest with a predominance of *Quercus* and *Pinus*, 06 November 2010, leg. F. Dovana, GDOR_5570; ibidem, 30 May 2019; Toscana, Grosseto, Monterotondo Marittimo, loc. Torrente Milia, forest with *Quercus suber* L. and *Pinus* sp., 05 December 2014, leg. R. Para, GDOR_5572; Emilia-Romagna, Ferrara, Mesola, forest with a predominance of *Pinus* spp. and *Quercus* spp., 03 December 2011, leg. A. Testoni, GDOR_5573. Spain: Aragona, under Quercus spp., 07 December 2014, leg. Adrián Hereza Ramón, GDOR_5574.

## 4. Discussion

The micromorphological analysis of the holotype of *Laccaria affinis* presented here has enabled the integration of information that was previously absent in Singer’s original description and in the subsequent study conducted by Mueller [2]. Our microscopic examination of small fragments of holotype material resulted in observations that are largely consistent with the findings reported by Singer [8] and Mueller [2], with the exception of the detection of rare, large cheilocystidia present at the lamella edge (Figure 7C). The examination of the stipitipellis has additionally uncovered the existence of caulocystidia variable in shape similar to those in *Laccaria affinis* f. *macrocystidiata* (=*Laccaria macrocystidiata*). This characteristic is regarded as a key diagnostic trait for the accurate identification of *Laccaria macrocystidiata* [13]. Many of the spores observed under the optical microscope had lost their ornamentation, but the analysis conducted under SEM provided a good view of the spore ornamentation. Numerous attempts to amplify the internal transcribed spacer (nrITS) region, as well as nrLSU and *RPB2* from fragments of the *L. laccata* var. *affinis* holotype, yielded no sequences attributable to the genus *Laccaria*. Dovana et al. [13] considered *L. macrocystidiata* to be an independent taxon at the species level. However, the authors did indicate the need for further study on the holotype of *L. affinis* to evaluate the true relationships between these two taxa. The morphological comparisons in Dovana et al. [13] relied on the existing literature [2, 8], which unfortunately did not provide data on the stipitipellis, a structure that the authors have identified as fundamental for the identification of *Laccaria macrocystidiata*. For this study, we personally obtained several collections of *Laccaria* during two expeditions to Kent conducted in November 2019. The first foray occurred in broadleaf woodlands dominated by oak and beech located just outside of the Bedgebury National Pinetum, while the second was conducted within the Bedgebury National Pinetum. In both habitats, numerous medium- to large-sized *Laccaria* basidiomes were found. Their morphology was deemed compatible with the macromorphological description of *L. laccata* var. *affinis* provided by Singer. In general, the basidiomata of *L. affinis* associated with the broadleaf woodland exhibited more orange colouration, and the surfaces of the pileus and stipe were characterised by more pronounced fine squamules or tufts. Conversely, the specimens from coniferous woodland typically displayed darker, brownish hues. However, the analysis of the nrITS sequences did not reveal any differences among the various morphotypes examined, which were collected from different habitats. Microscopically, the Kent basidiomata of *L. affinis* showed (i) globose spores with conical spines with a base smaller than 1 μm, (ii) the presence of cheilocystidia and (iii) caulocystidia similar to those observed in the holotype of *L. affinis*. The presence of cheilocystidia was consistent in all collections, although their dimensions and abundance varied among the different collections and within different analysed lamellae. In our phylogenetic analysis, the/L. affinis clade occupies an independent position among the Northern Hemisphere *Laccaria* species. The minimal differences between the nrITS sequences of the epitype of *L. affinis* and *L. macrocystidiata*, along with the results of the multilocus phylogenetic analysis, do not support the independence of these two taxa at the species level. The sequences previously identified as *L. macrocystidiata* (including the epitype) grouped within the/Laccaria macrocystidiata subclade A (as referenced in Dovana et al. [13]) within/L. affinis clade; however, no consistent morphological differences were found between the specimens belonging to this subclade and the other collections of *L. affinis*. The sample type of *L. laccata* var. *affinis* did not yield sequence data despite employing various extraction and sequencing methods. To address this shortcoming, we propose the formal designation of an epitype for *L. affinis*, collected from Bedgebury National Pinetum in Kent (GDOR_5565). This epitype is based on morphologically typical basidiomata that fit with the description provided by Singer [8]. Additionally, the designation is supported by micromorphological analyses conducted on the holotype of *L. affinis*, which further corroborates the morphological characteristics of the proposed epitype. The molecular characterisation of the epitype is well-defined by nrITS and *RPB2* sequences, providing a robust framework for future studies. *Laccaria affinis* is widespread in Europe, North Africa and Türkiye, very variable in morphology but characterized by generally medium-sized to large orange to brown basidiomata, light pink gills in young specimens and a squamulose to fibrillose-scaly pileus. The stipe is at least partially whitish furfuraceous in upper part (not very noticeable under moist conditions), with white mycelium at the base of the stipe, globose to subglobose spores with highly variable spines up to 2.5 μm in length, and lastly, cheilocystidia (more or less abundant in different basidiomata, sometimes difficult to observe in herbarium specimens) and caulocystidia are present. *Laccaria laccata*, another species with four-spored basidia that can share the same habitat as *L. affinis*, differs because of its smaller basidiomata, with a pileus that does not exceed 3 cm in diameter, smaller spores (7.5–8.8 × 5.7–6.4 μm) from subglobose to broadly ellipsoid, smaller cheilocystidia [8] and no caulocystidia (personal observation). *Laccaria populina* Dovana, recently described from Italy, could be confused with *L. affinis*. It differs in the glabrous to fibrillose, not furfuraceous stipe, absence of larger cheilocystidia, stipitipellis without caulocystidia, and association with Salicaceae [60]. The first collected basidiomes of *L. albifolia* were identified as *Laccaria affinis* based on the monograph on the genus *Laccaria* in Italy by Contu [12]. However, some macro- and micro-morphological differences prompted us to conduct a molecular study to clarify the relationships between these collections and *L. affinis*. Phylogenetically, *Laccaria albifolia* belongs to the/L. bicolor clade that mainly includes species with violet to purple lamellae and violet basal mycelium, but both these features are neither present in *L. albifolia* nor in *L. araneosa* (belonging to the same clade)*. Laccaria albifolia* is characterised by its minute basidiomata, pectinate pileus considerably paler towards the margin than in the centre, lamellae generally initially whitish, spores on average from subglobose to broadly ellipsoid with conical spines (up to 1.2 μm in length and less than 1 µm in width) and presence of cylindrical to irregular flexuose cheilocystidia and pleurocystidia. The white gills in young basidiomata and the darker colour of the stipe compared to that of the pileus margin are two features that facilitate the macromorphological identification of this species. Our Italian collections of *L. albifolia* primarily originated from xerophilous forests in Mediterranean areas, characterised by the presence of Fagaceae (oaks and beeches) and *Pinus* spp. The six nrITS sequences retrieved from GenBank nested within/L. albifolia clade, and on the base of the nrITS region, belong to the same species and were from Hungary, Portugal and Spain, where they are associated with different species of oaks (*Quercus ilex*, *Q. petraea* (Matt.) Liebl., *Q. rotundifolia* Lam. and *Q. suber*). Morphologically and phylogenetically, *L. albifolia* is closely related to *L. araneosa*; however, the latter is distinguished by its orange-brown lamellae, predominantly globose or subglobose spores (Qav = 1.03), the absence of cheilocystidia and pleurocystidia, and a distinct geographical distribution in temperate Korean forests [50]. *Laccaria laccata* var. *pallidifolia* is close to *L. albifolia*, but it differs due to the presence of pale orange or pink lamellae, pale orange stipe, spores with longer spines up to 2 μm and different phylogenetic positions (Figure 1) [14].

## Data Availability

The original contributions presented in this study are included in the article/Supplementary Material. Further inquiries can be directed to the corresponding authors.

## Supplementary Materials

The following supporting information can be downloaded at: https://www.mdpi.com/article/10.3390/jof11010011/s1, Table S1: *Laccaria* collections used in phylogenetic analyses. Sequences in bold were newly generated for this study.
