# Supplementary material for: Description of the New Species Laccaria albifolia (Hydnangiaceae, Basidiomycota) and a Reassessment of Laccaria affinis Based on Morphological and Phylogenetic Analyses
Source: J Fungi (Basel). 2024 Dec 27;11(1):11. doi: 10.3390/jof11010011 (PMC11766927; doi:10.3390/jof11010011)
Supplement: Supplementary file 1 [file jof-11-00011-s001.zip › jof-3382393-supplementary.pdf]

Table S1. *Laccaria* collections used in phylogenetic analyses. Sequences in bold were newly generated for this study.

Taxa	Specimens	Locality	GenBank Accession number				References
			ITS	LSU	tef	rpb2	
L. acanthospora	AWW485 (holotype)	China: Tibet	JX504102	JX504186	KU686073	KU685916	[15, 39]
L. aff. *angustilamella*	GMM6171	China	JX504132	_	_	_	[39]
L. affinis	GMM7618	France	KM067852	_	_	_	[40]
L. affinis	GMM7619	France	KM067853	_	_	_	[40]
L. affinis	GMM7617	France	KM067851	_	_	_	[40]
L. affinis	GMM7602	France	KM067842	_	_	_	[40]
/L. affinis clade *							
Laccaria sp. *	SB2151	Portugal	JX504173	JX504250	MT436063	MT431176	[15, 39]
Laccaria sp. *	R97C1	Denmark	OM431939	_	_	_	Direct Submission
Laccaria sp. *	E88B1	Denmark	OM431999	_	_	_	Direct Submission
Laccaria sp. *	E79A11	Denmark	OM431951	_	_	_	Direct Submission
Laccaria sp. *	H21570	Tunisia	KU973854	_	_	_	[41]
Laccaria sp. *	kz14	Germany	EF372410	_	_	_	[42]
L. laccata *	SB2160	Portugal	KM067889	_	_	_	[40]
L. laccata var. *moelleri* *	MoeFr	France	GQ406465	GQ406498	_	_	[43]
L. laccata *	ECC16112810	Spain	MW376680	_	_	_	Direct Submission
L. affinis *	GDOR5561 KENT	England: Kent	**PQ642692**	_	_	_	this study
Uncultured *Laccaria* *	clone MT4	unknown	MT730594	_	_	_	Direct Submission
L. proxima *	GMM7038	Russia: Caucasus	MT279221	MT279202	MT436064	MT431177	[5]
L. affinis *	GDOR5562 KENT	England: Kent	**PQ642693**	_	_	_	this study
Uncultured ectomycorrhiza *	clone C8	Denmark	AM159600	_	_	_	[44]
L. affinis *	GDOR5564 KENT	England: Kent	**PQ642691**	_	_	_	this study
L. affinis *	GDOR5563 KENT	England: Kent	**PQ642689**	_	_	_	this study
Uncultured *Laccaria* *	H12IK04	Germany:Thuringia	HF675632	_	_	_	Direct Submission
Uncultured *Laccaria* *	Sp 1-891-798	Denmark	AM161523	_	_	_	[44]
L. affinis *	GDOR5565 (epitype) KENT	England: Kent	**PQ642690**	_	_	**PQ653981**	this study
/L. affinis subclade A (includes holotype of L. marcocystidiata) **							
L. macrocystidiata **	GDOR_5076	Italy	MW584898	_	_	_	[13]
L. macrocystidiata **	GDOR_ 5077	Italy	MW584897	_	_	_	[13]
L. macrocystidiata **	GDOR_5084	Italy	MW584896	_	_	_	[13]
L. macrocystidiata **	GDOR_5083	Italy	MW584894	_	_	_	[13]
L. macrocystidiata **	AH38993	Italy	MW751684	_	_	_	[13]
Laccaria sp. **	LM4966	Hungary	KM576421	_	_	_	[45]
Uncultured *Laccaria* **	clone 189	unknown	KC686872	_	_	_	[46]
L. macrocystidiata var. *longispinosa* **	GDOR_5081	Italy	MW584889	MZ267722	_	_	[13]
L. macrocystidiata **	AH38995	Italy	MW751683	_	_	_	[13]
L. macrocystidiata **	AH38996	Italy	MW751682	_	_	_	[13]
L. macrocystidiata **	SOMF30438	Greece	OM286886	_	_	_	Direct Submission
Uncultured fungus **	clone AL-A05	Switzerland	KX886047	_	_	_	[47]
L. cf. *laccata* **	Montri-216	unknown	MK028444	_	_	_	[48]
Uncultured Tricholomataceae **	clone MT10	unknown	MT730600	_	_	_	Direct Submission
L. cf. marittima **	clone M49	Germany	EU816633	_	_	_	[49]

L. affinis **	GDOR5568	Italy	**PQ642687**	_	_	_	this study
L. affinis **	GDOR5567	Italy	**PQ642688**	_	_	_	this study
L. affinis **	GDOR5566	Italy	**PQ642686**	_	_	_	this study
L. macrocystidiata **	GDOR5075 (epitype)	Italy	MW584890	_	_	_	[13]
L. alba	GMM6131	China	JX504131	JX504210	KU686079	KU685930	[15, 39]
L. alba	KUN 20120807-69	South Korea	MG519542	MG519583	MG551649	MG551616	[50]
L. alba	AWW438	China	JX504094	JX504178	KU686072	KU685912	[15, 39]
L. albifolia clade ***							
L. laccata ***	SB2214	Portugal	KM067891	_	_	_	[40]
Uncultured fungus ***	EB178	Portugal	GQ205354	_	_	_	[51]
Uncultured mycorrhizal fungus ***	557 Laccaria	Portugal	FJ897201	_	_	_	[52]
L. sp. ***	LM5641	Hungary	KM576423	_	_	_	[45]
L. sp. ***	SB2067	Portugal	JX504171	JX504248	_	_	[39]
Uncultured fungus ***	H0305	Spain	MG274054	_	_	_	[53]
L. albifolia ***	GDOR 5570	Italy	**PQ642681**	**PQ642695**	_	_	this study
L. albifolia ***	GDOR 5571	Italy	**PQ642682**	**PQ642697**	_	_	this study
L. albifolia ***	GDOR 5572	Italy	**PQ642685**	**PQ642698**	_	_	this study
L. albifolia ***	GDOR 5573	Italy	**PQ642683**	**PQ642696**	**PQ653978**	**PQ653980**	this study
L. albifolia ***	GDOR5569 (holotype)	Italy	**PQ642680**	**PQ642694**	**PQ653979**	_	this study
L. albifolia ***	GDOR 5574	Spain	**PQ642684**	_	_	_	this study
L. amethysteo-occidentalis	AWW557	USA: California	MT279220	MT279200	MT436061	MT431174	[5]
L. amethysteo-occidentalis	AWW556	USA: California	JX504107	JX504191	_	KU685919	[15, 39]
L. amethystina	GMM7633	France	JX504154	JX504228	_	_	[15]
L. amethystina	GMM7621	France	JX504150	JX504224	KU686152	KU686046	[15, 39]
L. anglica	AngFr	France	GQ406459	_	_	_	[43]
L. angustilamella	HKAS41483 (holotype)	China	_	JX504233	_	_	[15]
L. araneosa	KNU20120912-40 (holotype)	Korea	MG519548	MG519588	MG551654	MG551621	[50]
L. araneosa	KNU20120912-25	Korea	MG519550	MG519590	MG551656	MG551623	[50]
L. araneosa	SFC20130917-21	Korea	MG519549	MG519589	MG551655	MG551622	[50]
L. aurantia	KUN-F 78557 (holotype)	China	JQ670895	_	_	_	[54]
L. aurantia	MB-FB-101109	China	JQ681209	_	_	_	[54]
L. bicolor	AWW585	USA: Oregon	JX504111	JX504194	_	_	[39]
L. bullipellis	AWW465 (holotype)	China: Tibet	JX504100	JX504184	_	KU685914	[15, 39]
L. dallingii	Corrales 543	Panama	MT279238	MT279213	MT436076	MT431187	[5]
L. dallingii	Corrales 571 (holotype)	Panama	MT279240	MT279214	_	_	[5]
L. diospyricola	CAL1771 (holotype)	India	MK776767	_	_	_	[55]
L. fagacicola	HKAS90435 (holotype)	China	MW540806	_	_	_	[56]
L. fagacicola	HKAS107731	China	MW540807	_	_	_	[56]
L. fengkaiensis	HKAS106739 (holotype)	China	MN585657	MN621238	_	_	[6]
L. fengkaiensis	HKAS106741	China	MN585658	_	_	_	[6]
L. fortunensis	Corrales 74 (holotype)	Panama	MT279246	_	_	_	[5]
L. fortunensis	Corrales 75	Panama	MT279247	_	_	_	[5]

L. fulvogrisea	KUN-F 78556 (holotype)	China	JQ670896	_	_	_		[54]
L. fulvogrisea	MB-FB-101105	China	JQ681210	_	_	_		[54]
L. gomezii	F1102433	CostaRica	_	MT279205	_	MT431180		[5]
L. gomezii	GMM7173	CostaRica	MT279227	MT279207	MT436071	MT431182		[5]
L. griseolilacina	SFC20190919-48 (holotype)	South Korea	MT322981	MT322983	MT333269	MT333266		[14]
L. himalayensis	AWW484 (holotype)	China: Tibet	JX504101	JX504185	_	KU685915		[15, 39]
L. himalayensis	AWW463	China: Tibet	JX504098	JX504182	_	KU685913		[15, 39]
L. indohimalayana	KD 17-46	India	MK575505	_	_	_		[57]
L. indohimalayana	KD 17-20 (holotype)	India	MK584157	_	_	_		[57]
L. japonica	TNS-F64167 (holotype)	Japan	KU962988	_	_	_		[58]
L. japonica	SFC20130928-07	Korea	MG519517	MG519565	MG551632	MG551594		[50]
L. laccata	GMM7586	Russia	KM067835	KU685859	_	KU686000		[15, 39]
L. laccata	GMM7020	Russia	KU685652	KU685795	_	KU685938		[15]
L. laccata	GMM7606	France	JX504147	JX504221	_	_		[39]
L. laccata	GMM7585	Russia	KM067834	_	_	_		[40]
L. laccata	GMM7587	Russia	KM067836	_	_	_		[40]
L. laccata var. *pallidifolia*	GMM7605	France	JX504146	KU685901	KU686154	KU686048		[15, 39]
L. laccata var. *pallidifolia*	Cripps 1370	USA: Montana	DQ149849	_	_	_		[14]
L. laccata var. *pallidifolia*	Cripps 1603	USA: Montana	DQ149851	_	_	_		[14]
L. laccata var. *pallidifolia*	Cripps 1633	USA: Montana	DQ149853	_	_	_		[14]
L. laccata var. *pallidifolia*	Cripps 1655	USA: Montana	DQ149847	_	_	_		[14]
L. laccata var. *pallidifolia*	Cripps 1724	USA: Montana	DQ149857	_	_	_		[14]
L. laccata var. *pallidifolia*	HMJAU26932	China	KM246792	_	_	_		Direct Submission
L. longipes	F1092175	USA: Michigan	KU685637	KU685780	_	_		[15]
L. longipes	MQ18R253-QFB30769	Canada	MN992191	_	_	_		Direct Submission
L. macrobasidia	HBAU15557	Korea	MW871602	_	_	_		Direct Submission
L. macrobasidia	SFC20170822-59	Korea	MT322982	MT322984	MT333268	MT333267		[4]
L. macrocystidia (?)	GMM7616	France	KM067850	KU685863	_	KU686004		[40, 15]
L. macrocystidia (?)	GMM7612	France	KM067847	KU685861	KU686122	KU686002		[40, 15]
L. macrocystidia (?)	GMM7626	France	KM067856	KU685865	KU686125	KU686006		[40, 15]
L. montana	M5464 (Isotype)	Switzerland	OR419935	_	_	_		[59]
L. aff. *montana*	AWW446	France	JX504097	JX504181	KU686054	KU686157		[40, 15]
L. montana / pumula complex	GMM7630tibet	China: Tibet	JX504151	JX504225	KU686009	KU686128		[40, 15]
L. moshuijun	HKAS93732	China	KU962989	_	_	_		[58]
L. moshuijun	MB-001113	China	KU962985	_	_	_		[58]
L. murina	ASIS24249	Korea	MG519552	MG519592	MG551658	MG551625		[50]
L. nanlingensis	GDGM94954 (holotype)	China	OR689442	OR785478	OR826273	OR835198		[7]
L. nanlingensis	GDGM84949	China	OR689441	OR785477	OR826274	OR835199		[7]
L. negrimarginata	BAP360	China: Tibet	JX504120	_	_	_		[39]

L. negrimarginata	GMM7631tibet	China: Tibet	JX504153	JX504227	KU686130	KU686011	[15, 39]
L. neovinaceoavellanea	GDGM52852 (holotype)	China	OR689447	OR785479	_	_	[7]
L. neovinaceoavellanea	GDGM53063	China	OR689448	OR785480	_	_	[7]
L. nitrophila	Corrales 467	Panama	MT279233	_	_	_	[5]
L. nitrophila	Corrales 595 (holotype)	Panama	MT279236	MT279211	MT436074	MT431186	[5]
L. nobilis	F1091206	USA: Michigan	KU685636	KU685779	_	_	[15]
L. nobilis	AWW584	USA: Oregon	JX504110	JX504193	_	KU685922	[15, 39]
L. oblongospora	OblFr	France	GQ406466	_	_	_	[43]
L. ochropurpurea	PRL4777	USA: Illinois	KU685733	KU685883	_	KU686025	[15]
L. ohiensis	KH_07192006_1	USA: Indiana	KU685720	KU685871	_	KU686014	[15]
L. ohiensis	GMM7028	Russia: Caucasus	KU685653	KU685796	_	KU685939	[15]
L. pallidorosea	KUN-HKAS53170	China	MW540809	_	_	_	[56]
L. pallidorosea	KUN-HKAS107730 (holotype)	China	MW540808	_	_	_	[56]
L. parva	SFC20120919-05 (holotype)	Korea	MG519529	MG519573	MG551640	MG551604	[50]
L. parva	SFC20120906-01	Korea	MG519527	MG519572	MG551639	MG551602	[50]
L. populina	GDOR411 (holotype)	Italy	MN871894	MN873018	_	_	[60]
L. populina	GDOR 408	Italy	MN871895	MN873017	_	_	[60]
L. prava	HKAS106742 (Holotpye)	China	MN585660	_	_	_	[6]
L. prava	HKAS106745	China	MN585661	_	_	_	[6]
L. proxima	308	Spain	MN663149	_	_	_	Direct Submission
L. proxima	GMM7584	Russia	KU685717	KU685858	KU686120	KU685999	[15]
L. pseudomontana	Cripps 1771	USA: Colorado	DQ149870	_	_	_	[14]
L. pseudomontana	Cripps 1625	USA: Colorado	DQ149871	_	_	_	[14]
L. pumila	GMM7637	France	JX504156	JX504229	KU686158	_	[15, 39]
L. pumila	GMM7636	France	KM067860	_	_	_	[40]
L. roseoalbescens	LM5099 (holotype)	Mexico	KJ874328	KJ874331	_	_	[61]
L. roseoalbescens	VB4678	Mexico	KJ590509	KJ590510	_	_	[61]
L. rubroalba	KUN-HKA 90753 (holotype)	China	KX449358	_	_	_	[62]
L. rubroalba	KUN-HKA 90766	China	KX449359	_	_	_	[62]
L. rufobrunnea	GDGM82878 (holotype)	China	OR689443	OR785482	OR826272	OR835197	[7]
L. rufobrunnea	GDGM89627	China	OR689444	OR785483	_	_	[7]
L. salmonicolor	GMM7596tibet	China: Tibet	JX504143	JX504218	KU686151	KU686045	[15, 39]
L. salmonicolor	GMM7602	China: Tibet	JX504145	JX504220	_	_	[39]
L. scotica	ScoFr	France	GQ406468	_	_	_	[43]
L. squarrosa	DM63 (holotype)	Mexico	MF669958	MF669965	_	_	[63]
L. squarrosa	DM121	Mexico	MF669960	MF669967	_	_	[63]
L. stellata	SYC 207 (paratype)	Panama	KP877339	_	_	_	[64]
L. stellata	Corrales 27	Panama	MT279231	MT279210	_	MT431185	[5]
L. striatula	1475	USA: New York	OQ612526	_	_	_	[65]
L. striatula	CNV105	unknown	MT345281	_	_	_	Direct Submission
L. torosa	SFC20150902-17 (holotype)	Korea	MG519561	MG519598	MG551664	MG551631	[50]
L. torosa	KA12-1306	Korea	MG519562	_	_	_	[50]
L. tortilis	ASIS22273	Korea	MG519533	_	_	_	[50]

L. tortilis	GMM7635	France	JX504155	KU685906	KU686156	KU686053	[15, 39]
L. trichodermophora	GMM7712	USA: Texas	KM067866	_	_	KU686012	[15, 40]
L. trichodermophora	GMM7733	USA: Texas	_	JX504230	_	KU686013	[15, 39]
L. trichodermophora	KH_LA06_013	USA: Louisiana	KM067881	KU685872	_	_	[15, 40]
L. trullisata	PRL7587	unknown	JX504170	JX504247	KU686153	KU686047	[15, 39]
L. trullisata	WCG2075	unknown	KM067894	_	_	_	[40]
L. umbilicata	GDGM82883	China	OR689445	OR785485	OR826270	OR835194	[7]
L. umbilicata	GDGM82911 (holotype)	China	OR689446	OR785486	OR826268	OR835192	[7]
L. versiforma	SFC20120926-01 (holotype)	Korea	MG519556	MG519594	MG551660	MG551627	[50]
L. versiforma	ASIS20939	Korea	MG519557	MG519595	MG551661	MG551628	[50]
L. vinaceoavellanea	SFC20150810-10	Korea	MG519539	MG519580	MG551646	MG551614	[50]
L. vinaceoavellanea	A2986	Korea	JN942810	JN939738	_	JN993520	Direct Submission
L. vinaceobrunea	F1110429	USA: Texas	_	KU685783	_	_	[15]
L. yunnanensis	MB-FB-001107	China	JQ670897	_	_	_	[54]
OUTGROUP							
L. ambigua	PDD 89696	New Zealand	KU685725	KU685876	KU686132	KU686018	[15]
